# Leptin Silencing Attenuates Lipid Accumulation through Sterol Regulatory Element-Binding Protein 1 Inhibition in Nasopharyngeal Carcinoma

**DOI:** 10.3390/ijms23105700

**Published:** 2022-05-20

**Authors:** Sheng-Dean Luo, Hsin-Ting Tsai, Tai-Jan Chiu, Shau-Hsuan Li, Ya-Ling Hsu, Li-Jen Su, Meng-Hsiu Tsai, Ching-Yi Lee, Chang-Chun Hsiao, Chang-Han Chen

**Affiliations:** 1 Department of Otolaryngology, Kaohsiung Chang Gung Memorial Hospital and Chang Gung University College of Medicine, Kaohsiung 83301, Taiwan; rsd0323@cgmh.org.tw (S.-D.L.); tiuy773@gmail.com (Y.-L.H.); 2 Graduate Institute of Clinical Medical Sciences, College of Medicine, Chang Gung University, Taoyuan 33302, Taiwan; chiutaijan@gmail.com; 3 Department of Medical Research, Chung Shan Medical University Hospital, Taichung 40201, Taiwan; thtsophia66@gmail.com (H.-T.T.); graduate@csmu.edu.tw (C.-Y.L.); 4 Institute of Medicine, Chung Shan Medical University, Taichung 40201, Taiwan; 5 Department of Hematology-Oncology, Kaohsiung Chang Gung Memorial Hospital and Chang Gung University College of Medicine, Kaohsiung 83301, Taiwan; lee0624@cgmh.org.tw; 6 Department of Biomedical Sciences and Engineering, National Central University, Taoyuan 32001, Taiwan; sulijen@gmail.com (L.-J.S.); billy1990914@gmail.com (M.-H.T.); 7 Education and Research Center for Technology Assisted Substance Abuse Prevention and Management, College of Health Science and Technology, National Central University, Taoyuan 32001, Taiwan; 8 Division of Pulmonary and Critical Care Medicine, Kaohsiung Chang Gung Memorial Hospital and Chang Gung University College of Medicine, Kaohsiung 83301, Taiwan

**Keywords:** leptin, NPC, SREBP1

## Abstract

Leptin is a crucial regulator of metabolism and energy homeostasis in mammals. Many studies have investigated the impacts of leptin on human cancers, such as proliferation and metastasis. However, the mechanisms underlying leptin-mediated regulation of lipid metabolism in nasopharyngeal carcinoma (NPC) remain incompletely understood. In the current study, leptin downregulation ameliorated lipid accumulation, triglyceride, and cholesterol levels. Mechanistically, diminished leptin by siRNA not only inhibited sterol regulatory element-binding protein 1 (SREBP1), a master regulator of lipid metabolism, at the mRNA and protein levels, but also reduced SREBP1 downstream target expressions, such as fatty acid synthase (FASN) and stearoyl-CoA desaturase-1 (SCD1), in NPC cells. In addition, leptin expression could modulate the promoter activity of SREBP1. We also found that pharmacological inhibition of poly-ADP ribose polymerase-γ (PPAR-γ) resulted in increased SREBP1 expression in leptin-depleted NPC cells. Functionally, SREBP1 overexpression overcame the effects of leptin-silencing attenuated triglyceride level, cholesterol level and cell survival in NPC cells. Taken together, our results demonstrate that leptin is an important regulator of lipid metabolism in NPC cells and might could be a potential therapeutic target for treatment of NPC patients.

## 1. Introduction

Metabolic reprogramming is a hallmark of cancer. Studies show that deregulated lipid metabolism is involved in cell proliferation, differentiation, and metastasis in several cancers, including nasopharyngeal carcinoma (NPC) [1,2,3,4]. In NPC, increased accumulation of lipid droplets in tumor tissue has been observed [4]. However, the molecular mechanism of lipid metabolism in NPC remains unclear and needs to be further investigated.

NPC is one of the most common head and neck cancers in East and Southeast Asia [5,6]. It arises from the mucosa of the nasopharynx and is associated with Epstein–Barr virus (EBV) latent infection, genetic factors, and exposure to carcinogens [7,8,9]. According to the histopathological classification proposed by the WHO, NPC is divided into three types: keratinizing squamous cell carcinoma, nonkeratinizing squamous cell carcinoma, and nonkeratinizing undifferentiated carcinoma. Nonkeratinizing squamous cell carcinoma is the major subtype of NPC in Taiwan. To date, although there has been improvement in therapy in recent years, high recurrence and metastasis remain the main problems in NPC patients, resulting in an overall poor prognosis [10]. Thus, it is necessary to further investigate the novel molecules that contribute to NPC progression to improve the diagnosis, management, and treatment.

Leptin, an adipocytokine, promote cancer progression through multiple mechanism, including various metabolic regulations in cancer cells [11,12,13]. Previous studies have shown that leptin regulates metabolism through an improvement of, and increase in, ATP production, as well as an increase in fatty acid oxidation [14,15,16]. However, the linkage between altered lipid metabolism and tumorigenesis by leptin and the underlying mechanism have not been completely evaluated in NPC.

Sterol regulatory element-binding protein 1 (SREBP1) is a key transcriptional factor that regulates the expression of genes that are important for lipid synthesis and uptake. For example, SREBP1 induces the expression of stearoyl-CoA desaturase (SCD1) and fatty acid synthase (FASN) to increase fatty acids synthesis [17,18,19]. Previous studies have shown that the expression of SREBP1 is increased in various cancers and it promotes tumor growth [20,21,22,23,24]. Latent membrane protein 1 (LMP1) activates SREBP1 to increase de novo lipogenesis in EBV-infected NPC cells [25]. Depletion of SREBP1 results in decreased cell viability in cancer cells [26,27]. However, whether SREBP1 is involved in leptin signaling remains an enigma.

In this study, we will investigate the relationship between leptin and lipid synthesis as well as the relative lipid synthesis genes in NPC cells. The results will provide a link between leptin and lipid metabolism disorder in NPC progression.

## 2. Results

### 2.1. Leptin Expression Is Involved in Lipid Accumulation and TG Content in NPC Cells

**Figure 1:**
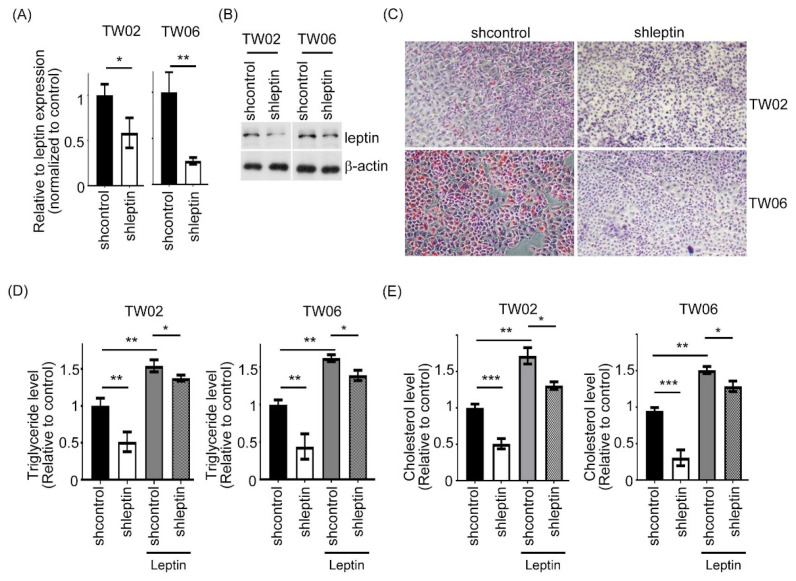
The effects of leptin on lipid accumulation and TG content in NPC cells. (**A**,**B**) The mRNA and protein levels of leptin were examined in leptin-depleted TW02 and TW06 cells and their corresponding control cells by QPCR and Western blotting. (**C**) The lipid accumulation was measured in leptin-depleted NPC cells. The intracellular TG content (**D**) and cholesterol content (**E**) in leptin-depleted NPC cells and negative control cells were investigated in the presence or absence of leptin (10 nM) stimulation. All data were obtained from three independent experiments. Data are presented as the mean ± SD. *: *p* < 0.05; **: *p* < 0.01; ***: *p* < 0.001.

Accumulating evidence indicates that cancer cells utilize lipid as an energy resource for maintaining cell growth [27,28]. To understand the potential involvements of leptin in the lipid synthetic process, we measured the lipid accumulation in NPC cells. First, leptin downregulation cells in both TW02 and TW06 cells were generated by the shRNA approach targeting the leptin (Figure 1A,B). As shown by Oil Red O staining, there is low accumulation of lipid droplets (LDs) in leptin-depleted TW02 and TW06 cells, as compared to the control cells (Figure 1C). Subsequently, we determined the intracellular triglyceride (TG) and cholesterol contents, which are mainly composed of LDs, and revealed that knockdown leptin significantly reduced the intracellular TG and cholesterol contents in both TW02 and TW06 cells (Figure 1D,E). However, both the TG and cholesterol levels were increased in leptin-depleted NPC cells upon addition of leptin to the culture medium (Figure 1D,E). Together, these data demonstrate that leptin expression was linked to lipid synthesis in NPC cells.

### 2.2. Leptin Regulates Lipid Metabolism in NPC Cells by Modulating SREBP1

**Figure 2:**
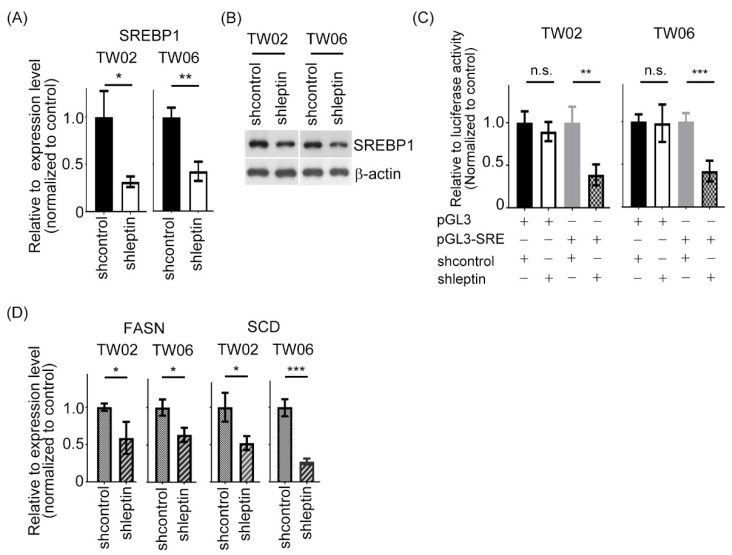
Leptin is involved in lipid metabolism in NPC cells. (**A**,**B**) The SREBP1 expression level was estimated by QPCR and Western blotting after leptin was silenced in TW02 and TW06 cells. (**C**) The luciferase assay was performed to determine the transcriptional activity of SREBP1 in leptin-depleted NPC cells and negative control cells. (**D**) The FASN and SCD1 at mRNA expression levels were estimated by QPCR after leptin was silenced in TW02 and TW06 cells. All data were obtained from three independent experiments. Data are presented as the mean ± SD. *: *p* < 0.05; **: *p* < 0.01; ***: *p* < 0.001; n.s.: not significant.

To further reveal the in-depth mechanism contributing to the leptin modulation of lipid accumulation in NPC cells, we first assessed the expression level of sterol regulatory element-binding protein 1 (SREBP1), a main factor that contributes to lipid metabolism. We discovered that leptin genetic silencing could result in downregulation of SREBP1 expression at both the mRNA and protein levels (Figure 2A,B). To further illustrate whether the transcriptional activity of SREBP1 could be modulated by leptin activity, a luciferase assay was performed. As shown in Figure 2C, the promoter activity of SREBP1 was significantly attenuated in leptin-knockdown TW02 and TW06 cells compared to their control, respectively. Many key enzymes, such as FASN and SCD1, have been reported to not only be involved in the process of lipid metabolism but also being downstream targets of SREBP1. Then, we evaluated whether their expressions were modulated by leptin in NPC cells. The results of the QPCR and Western blotting assays demonstrated that leptin silencing resulted in obvious downregulations of FASN and SCD1 at both the mRNA expression levels (Figure 2D). In view of these findings, we propose that leptin modulates the lipid metabolic genes by governing SREBP1 expression in NPC cells.

### 2.3. Leptin Modulates the SREBP1 Pathway via PPAR-γ in NPC Cells

**Figure 3:**
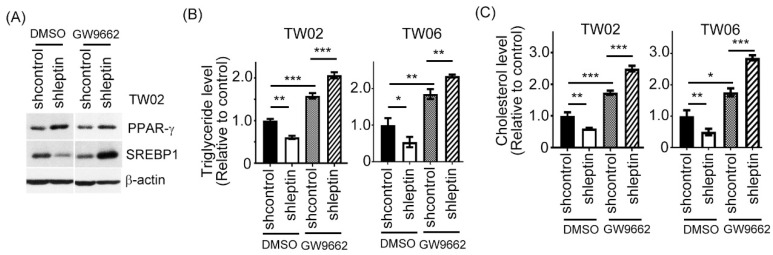
Leptin deficiency decreases SREBP1 via upregulation of PPAR-γ in NPC cells. (**A**) Western blot analysis of PPAR-γ and SREBP1 in leptin-depleted NPC cells in the absence or presence of GW9662. (**B**,**C**) The levels of TG and cholesterol in the indicated NPC cells with GW9662 treatment were measured. All data were obtained from three independent experiments. Data are presented as the mean ± SD. *: *p* < 0.05; **: *p* < 0.01; ***: *p* < 0.001.

Increasing evidence reports that PPAR-γ plays a key role in the onset of lipid metabolic syndrome [29]. In addition, PPAR-γ activation can decrease SREBP expression to prevent lipid accumulation [30]. On note, PPAR-γ has an anti-tumor effect in NPC [31]. This raises a hypothesis that SREBP1 inhibition in leptin-abolished NPC cells may be via PPAR-γ activation. We treated cells with the PPAR-γ antagonist GW9662 in leptin-silencing NPC cells and determined the expression profiles of PPAR-γ and SREBP1. By Western blotting assay, the PPAR-γ protein level was increased in leptin-silenced TW02 and TW06 cells. However, the inhibition of PPAR-γ by GW9662 in leptin-silencing cells clearly increased the protein expression level of SREBP1 (Figure 3A). We also assessed the intracellular TG and cholesterol levels in leptin-knockdowned TW02 and TW06 transfectants treated with GW9662. Expectedly, both the intracellular TG and cholesterol contents were increased in leptin-depleted cells after the administration of GW9662 (Figure 3B,C). Altogether, these data indicate that leptin regulates the SREBP1 pathway via PPAR-γ, thereby influencing the lipid accumulation in NPC cells.

### 2.4. Leptin Induces the Tumor Growth and Lipid Accumulation of NPC Cells Partially by Regulating SREBP1

**Figure 4:**
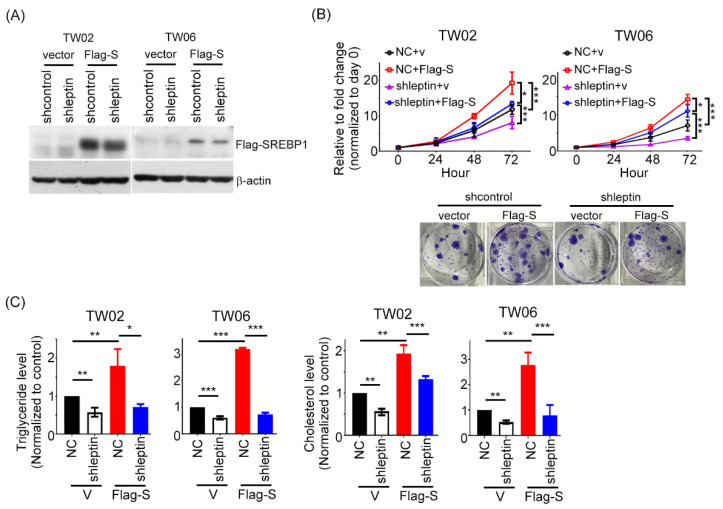
SREBP1 expression contributes to leptin-elicited NPC cell proliferation and lipogenesis. (**A**) Western blotting analysis of SREBP1 expression levels in shleptin-TW02 and shleptin-TW06 cells transfected with a vector alone or SREBP1 plasmid. (**B**) The CCK8 and colony formation assays determined the cell growth ability of leptin-silenced TW02 with SREBP1 expression or vector alone. (**C**) The levels of intracellular TG and cholesterol in shleptin-TW02 and shleptin-TW06 cells with SREBP1 expression were determined. v: vector; NC: shcontrol; Flag-S: Flag-SREBP1. All data were obtained from three independent experiments. Data are presented as the mean ± SD. *: *p* < 0.05; **: *p* < 0.01; ***: *p* < 0.001.

To further examine the role of leptin and SREBP1 in NPC cells, we performed rescue experiments. Our data showed that gain-of-function of SREBP1 in leptin-depleted NPC cells were established (Figure 4A). In addition, we found that overexpression of SREBP1 partially reversed the leptin downregulation-mediated inhibition of NPC cell proliferation and colony formation (Figure 4B). Notably, overexpression of SREBP1 partially rescued the impact of shleptin on lipogenesis in NPC cells (Figure 4C). These results demonstrate that leptin exerts its oncogenic function in NPC cells by depending on SREBP1 expression. 

### 2.5. Leptin Deficiency Decreases SREBP1 Level In Vivo

**Figure 5:**
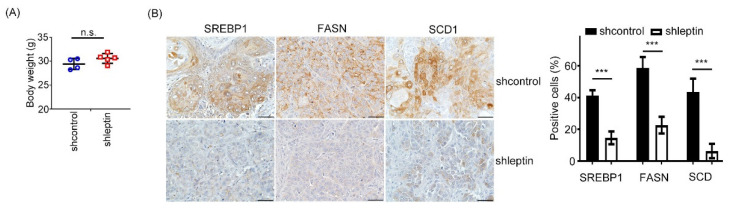
Leptin inhibition attenuates SREBP1 expression in vivo. (**A**) Mouse body weights were measured in shcontrol and shleptin groups. (**B**) IHC was performed to investigate the protein expression levels of SREBP1, FASN, and SCD1 in shcontrol and shleptin xenograft tumors. The quantifications of IHC staining are shown. The data are presented as the mean ± SD. ***: *p* < 0.001; n.s.: not significant.

We reasoned that leptin depletion suppressed the NPC tumorigenesis by impairing SREBP1 in vivo. Our data showed that the mouse’s body weight had almost no changes in the xenograft model (Figure 5A). Next, we conducted an IHC assay in shleptin-silencing xenograft tumor tissues to validate the expression patterns of SREBP1, FASN, and SCD1. As anticipated, the SREBP1, FASN, and SCD1 protein expression levels significantly declined as leptin was downregulated (Figure 5B), which are consistent with the in vitro findings. Collectively, our data provide in vivo evidence to support that leptin is essential for SREBP1 expression and NPC progression.

## 3. Discussion

A growing body of evidence shows that metabolic reprogramming is a recognized hallmark of cancer. Dysregulation of lipid metabolism contributes to tumor progression, including NPC. Leptin is upregulated in many cancers; however, the underlying lipid metabolic mechanisms of leptin on NPC progression are still unclear. In the current study, we discovered that lipid accumulation, such as lipid droplets, cholesterol, and triglycerides, were dramatically decreased by loss of leptin in NPC cells. Leptin inhibition could suppress SREBP1 as well as its downstream targets, FASN and SCD1 expressions, in vitro and in vivo. In addition, gain-of-function of SREBP1 in leptin-diminished NPC cells reversed shleptin-attenuated cholesterol level, triglycerides level, and growth ability. Furthermore, SREBP1 was dramatically increased in leptin-silencing NPC cells treated with PPAR-γ antagonist. Overall, these results indicate that leptin reprograms the lipid metabolism of NPC cells and might be a potential therapeutic target against NPC.

Recently, emerging evidence revealed that dysregulation of lipid metabolism contributes to disease development, including cancer [2]. Lipogenesis, such as lipid uptake and storage, is significantly elevated in malignant tumors and support tumor rapid proliferation, survival, and metastasis [32,33,34,35]. A typical principle for increased lipids in cancer cells are required for cell membrane synthesis and energy production. Recently, many studies reveal that lipids also could drive many signaling pathways to alter the components of the cell membrane, lipid biosynthesis and degradation, as well as cholesterol and triglycerides levels of cancer cells, promoting cancer cells with more malignant phenotypes [36]. In NPC, LMP2A, an Epstein–Barr virus (EBV)-encoded protein, can enhance lipid droplet formation and mediates metabolic-related genes to rewire the lipid metabolism pathways [4]. TINCR, a lncRNA, is an unfavorable prognostic factor in NPC and mediates the lipogenesis pathway to contribute to cancer progression in NPC [37]. Lipid turnover, fatty acid trafficking, and oxidation activation are observed in radiation-resistant NPC cells [38]. Altogether, this information implies that to explore the lipid metabolic signatures is crucial research to understand NPC progression. In other words, insight into the lipid metabolism of NPC will uncover a new therapeutic option for the treatment of patients.

Leptin, a non-glycosylated hormone, is necessary for food intake, energy homeostasis, hormone synthesis, hematopoiesis, and immunity [39,40]. Many studies report that leptin participates in the pathophysiology of neurovascular disease, obesity, endocrine disease, metabolism-related disease, and cancer [41]. In human cancers, a high leptin level is involved in accelerating tumor growth, such as breast, pancreatic, colorectal, ovarian, lung, esophageal, and gastric cancers, in cell and in animal models [41,42]. In line with previous studies, here, we demonstrated that leptin silencing by leptin targeting shRNA led to tumor growth attenuation in NPC cells. Our unpublished data showed that knockdown of endogenous leptin led to tumor growth inhibition in a xenograft model (manuscript under review). In this manuscript, we demonstrated that, in NPC cells, silencing leptin reduced the levels of lipid droplets, cholesterol, and triglycerides, thus suggesting that leptin has roles in regulating lipid metabolism in NPC. Substantial literature suggests that leptin plays a key role in cancer progression inhibition, called the “leptin paradox”. For instance, leptin is inversely related to breast cancer risk in premenopausal women [43]. High leptin expression is associated with a better outcome of colorectal cancer patients [44]. In preclinical models, such as pancreatic and HCC cells, the cell growth ability is inhibited under leptin stimulation [45,46]. Several lines of evidence show that leptin decreases the size of adipose tissue depots and the lipid content in skeletal muscle and liver tissue [47,48,49]. In addition, leptin increases the suppressive effects of insulin on hepatic metabolism and lipogenesis in white adipocytes [50]. Taken together, further investigations are needed to fully understand the complete mechanisms by which leptin functions in different cell contexts.

SREBP1, a major transcriptional factor, directly regulates lipogenic genes for controlling the lipid metabolism in cells. Activated SREBP1 translocates from the cytoplasm to the nucleus and binds to the SRE (sterol regulatory element) cis-acting elements in the promoter region to trigger gene expression. FASN and SCD1 are downstream target genes of SREBP1 and have been reported to be metabolic oncogenes [51,52]. In NPC, elevated FASN expression was associated with aggressive disease and poor survival in NPC patients [22]. A growing body of evidence have reported that elevated SREBP1 at both the RNA or protein levels is not only associated with malignant transformation, cancer progression, and metastasis, in vitro and in vivo, but also with a poor survival outcome in many solid tumors [53]. These data indicated that SREBP1 expression plays a crucial role in cancer development and lipid metabolism. In this study, we found that SREBP1 expression was regulated by leptin in NPC cell. In addition, overexpression of SREBP1 in leptin-diminished NPC cells reversed shleptin-inhibited tumor proliferation in vitro and in vivo as well as the intracellular TG, cholesterol, and phospholipid levels, indicating that leptin induced tumor growth and lipid metabolism are required for SREBP1 expression.

In conclusion, we demonstrated that leptin contributed to lipid metabolic reprogramming in NPC via modulating SREBP1 expression and SREBP1-mediated downstream targets. SREBP1-mediatd lipogenesis is essential for leptin-regulated NPC cell growth in vitro and in vivo. Of note, PPAR-γ participated in leptin/SREBP1 signaling. Taken together, our findings provide novel insights into the mechanism of leptin-mediated lipid metabolism and NPC progress through a SREBP1-dependent manner. These results may help to develop a strategy of treatment and valuable therapeutic drugs for NPC patients in the future.

## 4. Materials and Methods

### 4.1. RNA Isolation, Reverse Transcription and RT-qPCR

Total RNA was extracted from NPC cells using TRIzol reagent (Invitrogen, Carlsbad, CA, USA). A PrimeScript™ RT reagent Kit (TAKARA) reverse transcription kit was used to reverse transcribe mRNA from NPC cells into cDNA. Real-time quantitative PCR was performed using SYBR Green of RT Master Mix (TAKARA). The primer sequences for human SREBP1, FASN, and SCD used in this study were SREBP1 forward: 5′-ACAGTGACTTCCCTGGCCTAT-3′; reverse: GCATGGACGGGTACATCTTCAA-3′; FASN forward: 5′-AAGGACCTGTCTAGGTTTGATGC-3′; reverse: 5′-TGGCTTCATAGGTGACTTCCA-3′; SCD1 forward: 5′-TCTAGCTCCTATACCACCACCA-3′; reverse: 5′-TCGTCTCCAACTTATCTCCTCC-3′ and GAPDH forward: 5′-GGAGCGAGATCCCTCCAAAAT-3′; reverse: 5′-GGCTGTTGTCATACTTCTCATGG-3′. Relative gene expression was calculated using 2^−^^ΔΔCt^ method.

### 4.2. Cell Culture and Cell Transfection

TW02 and TW06 human NPC cell lines (provided by Dr. Chin-Tarng Lin, National Taiwan University, Taiwan) were cultured in Dulbecco’s modified Eagle’s medium (DMEM) medium (Gibco, Franklin, TN, USA) supplemented with 10% fetal bovine serum and 100 U/mL penicillin and streptomycin (Gibco, Franklin, TN, USA). All cells were cultured in an atmosphere of 5% CO_2_ at 37 °C. The PCR-amplified human full-length SREBP1 cDNA was constructed into the mammalian cell expression vector pEntry (OriGene Technologies, Rockville, MD, USA). Plasmids were transfected into TW06 and TW02 cells using Lipofectamine 3000 (Invitrogen, Carlsbad CA, USA) according to the manufacturer’s instructions. For the loss-of-function study, the shRNA system was used to knock down the expression of leptin in NPC cell lines. Leptin-specific shRNA (5′-CUGACUCCUCUAAGCCACU-3′) and scramble shRNA were constructed into the pLKO vector and then transfected into NPC cells using Lipofectamine 3000 (Invitrogen, Carlsbad CA, USA) according to the manufacturer’s instructions. The knockdown efficacy of the leptin protein by the shRNA approach was determined by Western blotting.

### 4.3. Western Blotting

Total proteins in each sample were extracted by using RIPA lysis buffer containing a protease inhibitor cocktail (Merck Millipore, Carrigtwohill, Ireland). The protein concentration in the lysate was quantified by a Bio-Rad protein assay (Bio-Rad Laboratories, Irvine, CA, USA). The extracted proteins were loaded onto SDS-PAGE and transferred onto polyvinylidene fluoride (PVDF) membranes (Millipore, New York, NY, USA). The membranes were blocked in a 1% solution of nonfat milk powder for 1 h at room temperature and then incubated at with primary antibodies at 4 degrees Celsius overnight. Immunoblotting was performed with an antibody against FLAG tag (1:1000, TA50011) purchased from OriGene Technologies. Antibody against β-actin (1:20,000, A5541) was purchased from Sigma-Aldrich (Sheboygan Falls, WI, USA). Antibody against leptin (1:1000, A1300) was purchased from ABclonal. Antibodies against FASN (1:2000, DF6106), SCD (1:2000, DF13253), SREBP1 (1:1000, AF6283), and PPAR gamma (1:1000, DF6073) were purchased from Affinity. Horseradish peroxidase-conjugated goat anti-rabbit and goat anti-mouse antibodies (PerkinElmer Inc., Waltham, MA, USA) were incubated for 1 h at room temperature. Signals were revealed with Immobilon Western Chemiluminescent HRP Substrate (Merck Millipore, Carrigtwohill, Ireland).

### 4.4. Immunohistochemical Staining

Sections were incubated with primary antibody at 4 °C overnight. After phosphate-buffered saline with 0.5 % Tween-20 (PBST) washing, slides were incubated with a peroxidase-labeled polymer conjugated to goat anti-rabbit IgG as secondary antibody for 30 min (Agilent Technologies, Inc., Santa Clara, CA, USA, DaKo Real EnVision Detection Systems Peroxidase/DAB, Rabbit/Mouse). The staining was visualized with diaminobenzidine (DAB) kit and slides were counterstained with hematoxylin (Sigma-Aldrich Co., Sheboygan Falls, WI, USA), dehydrated, and mounted. A 200-fold field of view was taken, and the IOD analysis was performed by Image Pro Plus 6.0 image analysis software. The percentage of positive cells was assigned a score from 0 (<10%), 1 (10–30%), 2 (30–50%), 3 (50–75%), or 4 (>75%), and the staining intensities within the respective subcellular locations were noted as 0 = negative, 1 = weak, 2 = moderate, or 3 = strong [54].

### 4.5. Cell Proliferation Assay

Cell viability was determined by CCK8 assay. TW02 or TW06 cells transfected with shcontrol or shleptin were seeded into 96-well plates at a density of 5000 cells/well in the presence or absence of 100 nmol GW9662, a PPAR-γ antagonist. At 24, 48, and 72 h, indicated cells were washed with PBS, and the medium was replaced with 180 μL of fresh medium plus 20 μL of a CCK-8 kit reagent. The cells were cultured for 3 h, and the optical density (OD) of each well was measured with a microplate reader (Molecular Devices, San Jose, CA, USA) at 450 nm. Each experiment was performed in triplicate.

### 4.6. Colony Formation Assay

Cells (500 cells/well) were plated in 6-well plates and incubated with complete medium for 10 days. Next, the cells were fixed by methanol and stained with 10% crystal violet solution for 10 min at room temperature. The colony number was counted with ImageJ software.

### 4.7. Luciferase Reporter Assay

In total, 5 × 10^4^ cells were seeded in 24-well plates and co-transfected with pGL3-SREBP1 promoter reporter plasmid and pRL-TK by Lipofectamine 3000 transfection reagent. After 24 h, the firefly and Renilla luciferase activities were determined with a dual-luciferase reporter system (Promega). The measurement was performed on a Microplate Reader.

### 4.8. Triglyceride and Cholesterol Measurement

The triglyceride or cholesterol level from NPC cells were measured using a Triglyceride Assay kit (ab65336) or Cholesterol Assay kit (K623; BioVision, Milpitas, CA, USA), following the manufacturer’s instructions.

### 4.9. Oil Red O Staining

Cells were seeded in 24-well plates and cultured with completed medium. After 24 h, cells were washed with PBS twice, and stained with Oil Red O (Sigma) solution for 10 min. Then, cells were counterstained with hematoxylin (Abcam, Cambridge, MA, USA) for 1 min. Images were taken by using a light microscope. The number of lipid droplets was analyzed with ImageJ software.

### 4.10. Xenograft Studies

The cells (1 × 10^7^ shcontrol or shleptin NPC cells) were subcutaneously injected into the right flanking sites of male NOD-SCID mice (*n*  = 4 in shcontrol group, *n*  = 5 in shleptin group, 4 weeks). After 4 weeks, the tumors were weighed and excised. The body weight of the mice and tumor size were measured per week. Tumor volumes were determined by external measurements and calculated according to the equation V = (W^2^ × L)/2 (V = volume, L = length and W = width). After 30 days, the mice were sacrificed, and the tumor weights were measured. All procedures were approved by the guidelines of the Institutional Animal Care and Use Committee at Kaohsiung Chang Gung Memorial Hospital.

### 4.11. Statistical Analysis

All statistical analysis was performed using GraphPad Prism 8. Comparisons of the groups were analyzed by Student’s *t*-test. All data are presented as the mean  ±  SD. A *p*-value less than 0.05 was considered to be significant.

## Data Availability

The data presented in this study are available on request from the corresponding author.
